# Inadvertent Intrathecal Administration of Local Anesthetics Leading to Spinal Paralysis with Lipid Emulsion Rescue

**DOI:** 10.5811/cpcem.2016.12.33046

**Published:** 2017-01-27

**Authors:** Amit Gupta, Gabrielle L. Procopio, Patrick H. Charles, Monica Hernandez, Ruchi Patel

**Affiliations:** * Hackensack University Medical Center, Department of Emergency Medicine, Hackensack, New Jersey; † Hackensack University Medical Center, Department of Pharmacy, Hackensack, New Jersey

## Abstract

Bupivacaine and ropivacaine are local anesthetics frequently used for interscalene nerve blocks, which are generally well tolerated; however, some complications include pneumothorax, Horner syndrome, nerve injury and cardiovascular toxicity from vascular injection. On rare occasions, it may be associated with spinal paralysis. While the treatment is mostly supportive, we report an unusual case of administering intravenous lipid emulsion (ILE) as part of resuscitative efforts to hasten neurological recovery from spinal shock.

## INTRODUCTION

Interscalene nerve block refers to the technique of anesthetizing the roots or trunks of the brachial plexus in the neck between the anterior and middle scalene muscles. It is a commonly used procedure to provide anesthesia or analgesia for surgery of the shoulder and upper arm with rare complications. Several case reports have reported complete spinal cord paralysis and cardiovascular collapse after this procedure due to inadvertent epidural or subdural introduction of local anesthetic(s).^1^–^3^ Treatment in these cases was primarily supportive.

Bupivacaine/ropivacaine are commonly used local anesthetics for peripheral nerve blocks, belonging to the amide group. They function by reversibly binding to the intracellular portion of voltage-gated sodium channels, thereby blocking sodium influx into nerve cells*,* which prevents depolarization and failure to initiate and propagate action potentials.^4^ This effect slows impulse conduction in the sinoatrial and atrioventricular nodes, the His-Purkinje system, and atrial/ventricular muscle, which can lead to its most feared complications of myocardial depression, refractory ventricular dysrhythmias, and possible cardiac arrest due to inadvertent cardiovascular administration.^5^ Local anesthetic toxicity (LAT) is often refractory to conventional therapy, but an effective antidote is 20% intravenous lipid emulsion (ILE), Intralipid®.

We report a case of a patient who had complete spinal shock leading to quadriplegia and cardiovascular collapse after an interscalene block.

## CASE REPORT

A 43-year-old male presented to the emergency department (ED) for evaluation of acute respiratory arrest. Prior to arrival, the patient was at an outpatient surgical center being prepared for a right shoulder arthroscopic surgery under regional anesthesia. The anesthesiologist injected 10 mL of bupivacaine and 30 mL of ropivacaine, of unknown concentrations, while attempting to perform an interscalene block. Soon thereafter, the patient complained of difficulty breathing and subsequently developed complete paralysis and respiratory arrest requiring emergent endotracheal intubation prior to hospital arrival. No additional medications were administered at the time of intubation.

Physical exam revealed dilated pupils nonreactive to light, complete flaccid paralysis and inability to withdraw from noxious stimuli. The patient immediately received two liters of 0.9% normal saline and a dopamine infusion, initiated at 5 mcg/kg/min, due to persistent bradycardia (heart rate of 44 bpm) and hypotension. The local poison control center was contacted for recommendations on potential LAT. The decision was made to administer 20% ILE, with a bolus of 125 mL (1.5 mL/kg) over one minute, about 90 minutes after presentation. Shortly after, propofol was initiated for post-intubation sedation at 10 mcg/kg/min. The remaining volume of ILE (125 mL) was then immediately infused over two hours at a rate of 0.013 mL/kg/min.

Approximately 30 minutes after the initiation of ILE, the patient began to regain brainstem reflexes and eye movement, which progressed to head nods, then movement of his upper extremities. In addition, during that time period, his blood pressure and heart rate improved to within normal parameters. Sixty minutes later, the patient was able to communicate by writing down responses to questions. One hour after that, he had full recovery and was extubated. The patient recovered completely without any evidence of neurological sequelae and was discharged 30 hours after presentation.

## DISCUSSION

Bupivacaine and ropivacaine are frequently used for interscalene nerve blocks, since it has shown to provide rapid and effective local anesthesia. Although interscalene blocks are generally well tolerated, on rare occasions they may be associated with severe complications. During an interscalene block, the nerves are anesthetized at the root level of the brachial plexus located at the interscalene groove. In a rare occurrence, intrathecal injection may track back along the plexus roots into the epidural space. Intrathecal injection can also occur from either needle misplacement through an intervertebral foramina or via injection into a dural cuff.^6^,^7^ Injection into either the external jugular vein or vertebral artery is also possible. The complications from LAT range from dyspnea, bilateral arm weakness and apnea to hemodynamic collapse and in this case paralysis.^8^,^9^

Although the treatment is mostly supportive while the drug “wears off,” we report an unusual case of administering ILE as part of our resuscitative effort to hasten neurological recovery. Because of general lack of familiarity with the pharmacokinetic alterations with intrathecal route and toxicity associated with these drugs, it was decided to institute lipid emulsion early in the course. Pharmacokinetic properties are dependent on the total dose, drug concentration, route of administration and vascularity of the administration site. Data primarily exist regarding pharmacokinetic parameters for intravenous and epidural routes; information is limited when these drugs are administered via intrathecal route. A study by Rose et al. would suggest plasma pharmacokinetic parameters for intrathecal route lie in between the intravenous and epidural routes.^10^

ILE is an extensively studied agent for the management of LAT and has been demonstrated to be an effective antidote in a number of case reports. In a recent study surveying 45 United States poison control centers, 89% and 96% of medical directors surveyed stated they would “always” or “often” recommend ILE for patients experiencing shock or cardiac arrest from bupivacaine, respectively.^11^ Although most data are limited to case reports, current evidence suggests potential benefit when given as salvage therapy in patients presenting with overdoses involving lipophilic molecules.^12^

While several mechanisms have been proposed for its effectiveness, the most widely accepted theory is the creation of a lipid sink to sequester lipid-soluble drugs and thus remove them from the site of toxicity. ILE may pull the drug out of the aqueous plasma, which bathes the tissue, and redistributes the drug away from the site of toxicity into an area with high lipid fat compartment of the plasma.

The safety of using ILE to treat overdoses is largely unknown due to limited reporting. Rare complications including laboratory interference, pancreatitis, and acute respiratory distress syndrome have been reported with ILE use.^13^,^14^ Additional complications associated with ILE are extrapolated from the extended use as a part of total parenteral nutrition, including hypertriglyceridemia, fat embolism, and infection.^15^–^17^

In this case, since the patient began to show signs of neurologic recovery 30 minutes from the initiation of the bolus infusion there was no need to continue lipid therapy infusion. It should be noted that another lipid-based product, propofol, was also administered concomitantly with ILE; however, this is manufactured in a 10% lipid emulsion. The pharmacodynamics of propofol and ILE acting synergistically, and whether they provide a larger lipid sink, are unknown.

In addition to the uncertainty surrounding the safety of ILE for this indication, the appropriate dosing has yet to be determined. The dosing protocol most widely reported in the literature consists of an intravenous bolus of 1 to 1.5 mL/kg administered as a bolus followed by an infusion of 0.25 to 0.5 mL/kg/min for a duration of 30–60 minutes or until hemodynamic recovery.^18^,^19^ The bolus may be repeated one or two times in the event of continued asystole. In our case, the local poison center recommendation for ILE was based on the assumption that the premixed Intralipid® bag was 500 mLs. Our patient received half the recommended total volume as the premixed bag in stock only contained 250 mLs and providers were instructed to give the remainder of the bag’s contents, after the bolus, over two hours. While the recommended bolus dose was administered to our patient, an infusion of 125mL was administered over two hours (0.013 mL/kg/min), which was lower than that suggested.

To our knowledge, this is the first case report documenting ILE administration for spinal shock due to intrathecal administered of local anesthetics. In light of these uncertainties, it is reasonable to administer ILE for reversal of local anesthetic-induced toxicity. In summary, intrathecal anesthetic toxicity treatment is primarily supportive and due to its safety profile, ILE rescue may be attempted.
